# Prognostic factors in elderly patients with breast cancer

**DOI:** 10.1186/1471-2482-13-S2-S2

**Published:** 2013-10-08

**Authors:** Alessandro Cappellani, Maria Di Vita, Antonio Zanghì, Andrea Cavallaro, Gaetano Piccolo, Marcello Majorana, Giuseppina Barbera, Massimiliano Berretta

**Affiliations:** 1 Department of Surgery, General and Breast Surgery Unit, University of Catania, Catania, Italy; 2 Department of Radiology, Mediterranean Institute of Oncology, Viagrande, (CT), Italy; 3 Department of Medical Oncology, National Cancer Institute, Aviano, Italy

**Keywords:** Breast cancer, Elderly, Prognostic factors, Biomarkers, Geriatric assessment

## Abstract

**Background:**

Breast cancer (BC) remains principally a disease of old ages; with 35-50% of cases occurring in women older than 65 years. Even mortality for cancer increases with aging: 19.7% between 65 and 74 years; 22.6% between 75 and 84 years; and 15.1% in 85 years or more.

The study was aimed to investigate specific predictive factors for elderly patients so to select the best way to treat and follow these patients.

**Methods:**

A search was performed on Medline, Embase, Scopus using the following Key words: Breast cancer, Breast neoplasms, Aged, Elder, Elderly, Eldest, Older, Survival analysis, Prognosis, Prognostic factors, Tumor markers, Biomarkers, Comorbidity, Geriatric assessment, Axilla, Axillary surgery. 3029 studies have been retrieved. Paper in which overall or disease free survival were not end points, or age class was not well defined, or the sample was too small, were excluded. At last 42 papers fulfilled the criteria.

**Results and discussion:**

Lack of screening and delay in diagnosis may be responsible for the minor improvement in survival observed in elderly respect to younger breast cancer patients. Predictive factors are the same and must be assessed with the same attention reserved to younger women.

**Conclusions:**

Most of elderly patient are fit to undergo standard treatment and can get the same benefits of younger women. Nevertheless it is possible that some older women with early breast cancer can be spared too aggressive treatments. Geriatric assessment and co-morbidities can affect the prognosis modifying surveillance, life expectancy and compliance to therapies. They can thus be useful to select the better treatment, either surgical or radio or hormone - or chemo-therapy.

## Background

Breast cancer (BC) remains principally a disease of old ages; with 35-50% of cases occurring in women older than 65 years.

In the period 2004-2008, the National Cancer Institute’s Surveillance Epidemiology and End Results (SEER) has reported that about 40% of BSs have been diagnosed in elderly women: 19.7% in women aged between 65 and 74 years; 15,5% in women between 75 and 84 years, and 5.65% in those aged 85 years and older. Thus, mortality for cancer increases with aging: 19.7% between 65 and 74 years; 22.6% between 75 and 84 years; and 15.1% in 85 years or more [1].

Similar results have also been reported by other researchers (Table 1) [2-4].

**Table 1:** Survival for age class.

Age class	50-69	70-74	75-79	80+
5-year survival	89	81	76	70
10-year survival	84	77	67	66

From Ali et al, British Journal of Cancer, 2011.

**Figure 1:**
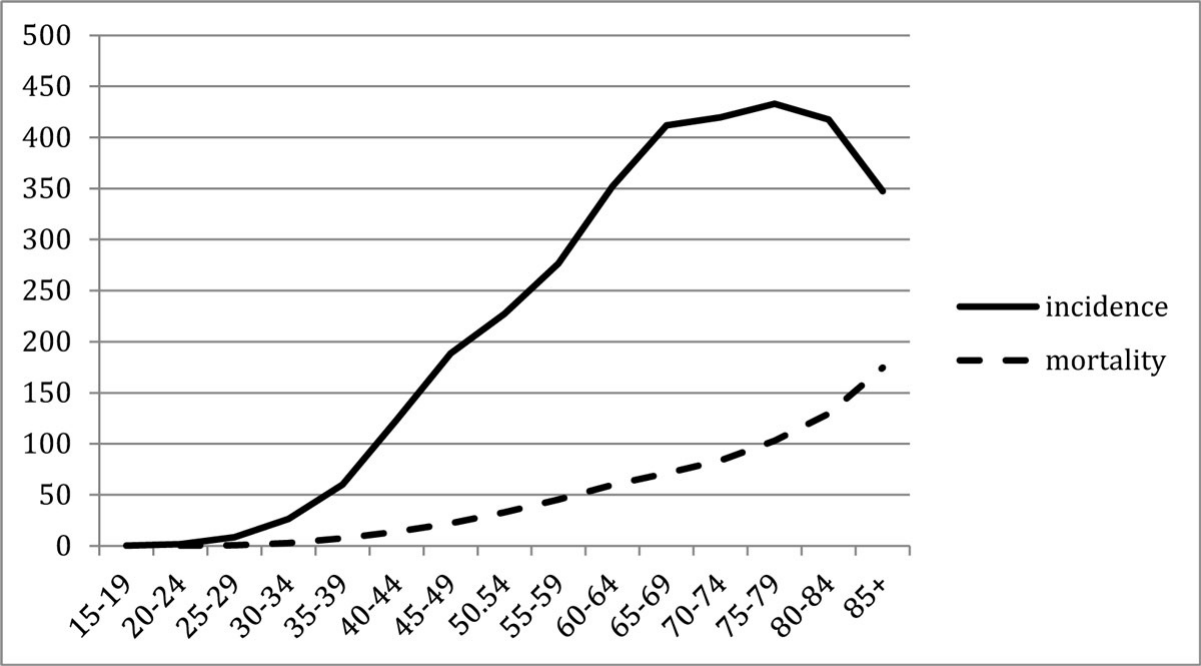
**Incidence ------- and death - - - - rates /100.000, 2005-2009 in USA, from SEER **[**1**]

**Figure 2:**
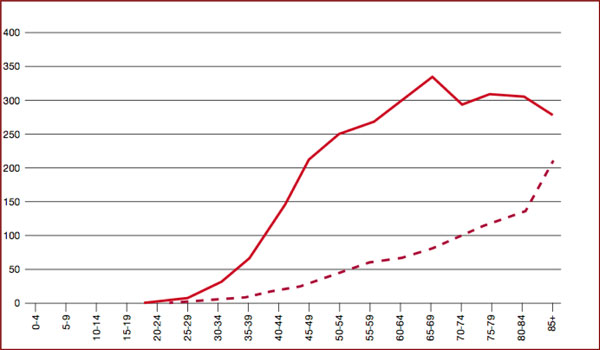
**Incidence -------- and death - - - - rates in Italy /100.000 (AIRTUM, 2006) **[**4**]

The improvement in the 5-year survival observed in the last decades in many countries (USA, GB, Italy, etc.) in all classes of ages (Figures 1, 2) [1-4] is less evident for older women [5]. Mortality for BC in USA has decreased by 24% between 1990 and 2000, mostly in younger women (3.3% per year), but less for older women (2% per year) [1].

In a Third World series, survival is even lower, related to the low life expectancy in these populations [6].

Are these results related just to a lack in screening, delayed diagnosis or to an under treatment?

In some study [7] the income influenced the probability for over- 65 to undergo screening regularly and therefore the prognosis, while a more recent study [8] on 173 women aged 80 years and older has showed a large percentage of early stages diagnoses: stage I (35%) and II (32.9%), with about 50% of cases showing negative axillary nodes. Less favorable are the data from Botteri [9] who has observed that the incidence of positivity lowered with the growing of age until 65 years, but increased thereafter. Thus, a recent study in post-menopausal women with Hormone Receptor positive breast cancer, reports a higher disease specific mortality with increasing age [10]. Other researchers have found a more frequent delay in diagnosis in older women [11], even if the influence of such delay cannot be definitely assessed [12].

To complicate the issue, most trials exclude women older than 70 years and, on the other side, is quite difficult to recruit these women in randomized trials, as demonstrated by the failure of a recent attempt [13].

Further, in some studies, the median follow up is just about two years and the recorded mortality is often not related to cancer (i.e., in the series of Solej, 8 out of 34 elderly patients died within two years, but only two deaths were cancer-related) [14], making impossible to distinguish the respective influence of the different prognostic factors.

While age is usually included among prognostic factors for BC, which prognostic factors should we consider in older women? Have such factors different value in older women respect to younger? Answering to such questions is the aim of the present paper.

## Methods

*Identification of key words*. Three series of key words (Table 2) were identified relating to the pathology (Breast cancer), to the age of patients and to prognostic factors.

**Table 2:** List of key words.

Breast cancer	Aged	Survival analysis
Breast neoplasms	Elder	Prognosis
	Elderly	Prognostic factors
	Eldest	Tumor markers
	Older	Biomarkers
		Comorbidity
		Geriatric assessment
		Axilla
		Axillary surgery

### Selection of studies

Reports investigating the role of prognostic factors in elderly patient with non metastatic breast cancer were selected for review using a search of PubMed, Embase, Scopus from 2006 to date April 30, 2012, using the lists of key words above indicated combined and crossed.

The search was limited to clinical trials, practice guidelines and review available in the English language. 3029 citations were retrieved. Papers without available abstract were excluded, Phase I, II, III pharmacological studies, were excluded and so were male breast cancer studies, case report or small series reports as well as psychological and behavioral studies. The remaining 2128 titles have been checked one by one leading to identify 243 possibly relevant papers.

The abstract or full text of all studies on breast cancer prognostic or predictive factors, risk of recurrence and survival have been reviewed to identify any relevant article. Eligible reports were those that examined either overall survival (OS) and/or disease free survival (DFS) in clinical series of primitive invasive breast cancer including over 70 years age patients; guidelines and review have been selected for relevant information and opinion. Articles where either OS or DFS were not used as clinical endpoints or where the correlation between prognostic factors and age was not evaluable were to be excluded from the review. Studies on samples smaller than 100 patients have been excluded as well. Nevertheless some articles from which it could be inferred any relevant information relating to epidemiology or management of elderly patient with breast cancer, have been retained too, even not fulfilling criteria.

Twenty-nine eligible studies were identified and further 32 retained for relevant information.

**Figure 3:**
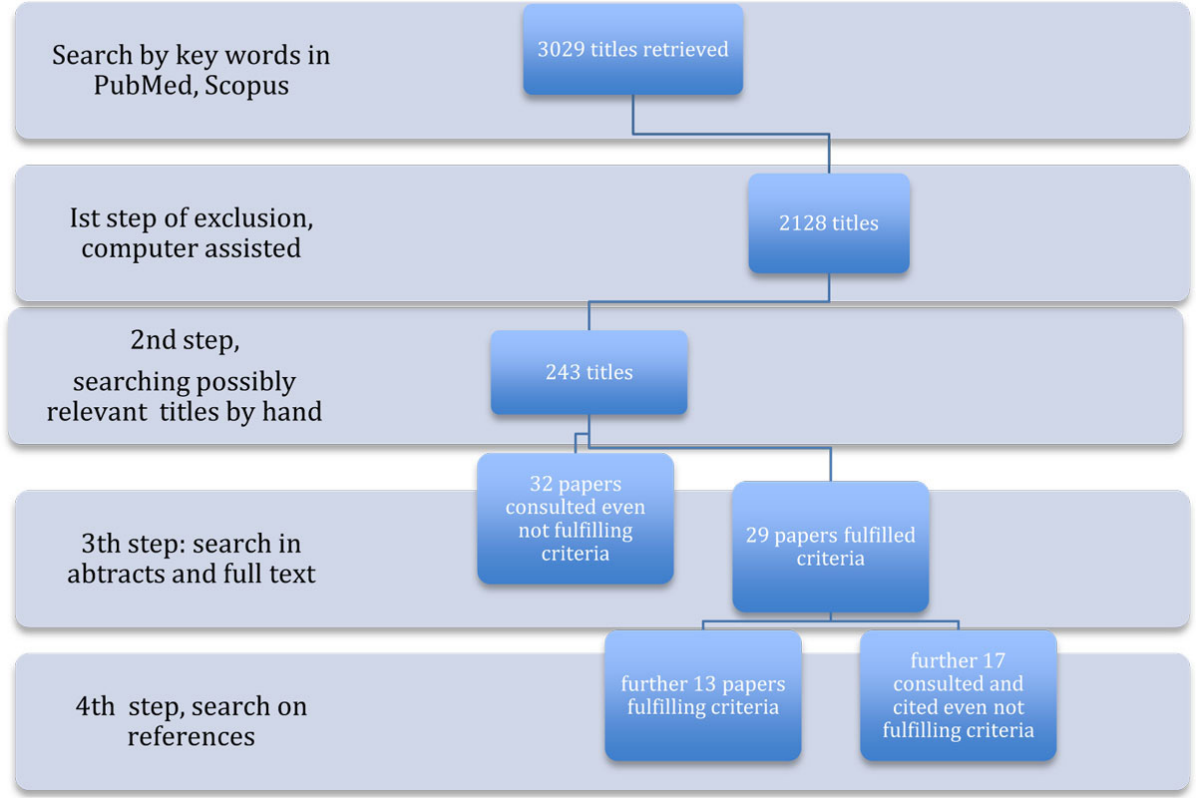
**Flow chart of the review**.

The bibliography of the reports was also searched by hand for every other important contribution retrieving further 30 papers (Figure 3). At last 42 papers fulfilled the criteria (See additional file 1) while 49 were consulted for relevant opinion even not fulfilling the criteria and are listed in additional file 2.

## Results and discussion

### Prognostic factors

The usual prognostic factors are the following:

- Axillary lymph node status;

- Tumor size;

- Age;

- Lymphatic/vascular invasion;

- Histologic grade;

- Histologic subtypes (e.g., tubular, mucinous [colloid], papillary);

- Response to neoadjuvant therapy;

- ER/PR status;

- HER2 gene amplification and/or over expression.

Comorbidities have been also included among prognostic factors as these can affect therapeutic approaches, both surgical and pharmacological [2,3,5,6,11,15]. Not all of these factors have been specifically evaluated regarding to the age

### Axillary lymph node status

Axillary nodal status is known to be the principal prognostic factor.

Many women in older age are deprived of this tool, mainly for three reasons: 1) Comorbidities affecting the possibility of adjuvant therapy; 2) a poor life expectancy; 3) avoidance of morbidity related to axillary surgery. Martelli et al, have studied the possibility to spare lymphectomy to older women in a randomized trial on 219 women aged 65-80, with early BC. They found just a 2% of clear axillary metastatic involvement at 5-years follow up [16]. A survey on the same sample at 15 years follow up showed no differences in OS between patients undergone axillary dissection (AND) and those who did not [17]. Only few studies include longer than 5-years follow up in patients with early stage cancer but, as already observed, it is difficult to find longer follow-ups in this class of age [1-9,11,12].

Albrand and Terret in their review agreed with the SIOG recommendation to treat axillary node in the elderly not differently than in younger women [18,19].

Since sentinel node biopsy (SNB) has been introduced, at least for suitable patients (women with breast cancer less than 3 cm in diameter), the axillary morbidity should no longer be a problem [20,21].

When SNB is positive for metastases, complete axillary (lymph) node dissection (AND) should be performed, even if metastasis in non sentinel nodes (NSN) are present in less than 50% of cases [20,22]. The treatment of axilla after a positive SNB remains controversial, The last recommendations of the International Society of Geriatric Oncology (SIOG) and European Society of Breast Cancer Specialists (EUSOMA) confirm that completion ALND for tumor-positive SLNB remains the standard of care but admit that omission of SLNB and completion ALND might be reasonable in some older patients [23-25]. Some Authors, in fact, felt that performing an AND in all cases would represent an overtreatment and that information from primitive neoplasm histology and from sentinel node could help to safely spare a not necessary AND. In 1999, Chu and Coll., in a cohort of women with a mean age of 52 years, after evaluating numerous possible predictive factors, found that only the size of primitive BC and of SN metastases were significative. They proposed not to perform AND in small primary BC (T1a, T1b) and SNB showing only micro metastases [26].

Further investigations demonstrated that the evaluation of various factors could provide a better definition of NSN probable metastases. MSKCC investigators have proposed a nomogram able to predict positivity of NSN after SNB positive for metastases, taking in account the use of frozen section, size, type and grade of BC, HR positivity number of SN positive and negative, quality of assessment of positivity (H&E, IHC). By this nomogram, even the smallest ductal GI, ER+ cancer, with SN positive only with IHC staining, shows a 6% of possibility of further metastases in NSN [27].

The nomogram has been tested also in different populations but only a few studies have confirmed its predictivity [28-35], still warning against the risk of underestimate the probability of additional metastases after the finding of micro metastases in the sentinel node. In 2005 the ASCO guidelines, as metastases are found in NSN in approximately 10% of patients with isolated tumor cells in the SLN and in 20% to 35% of patients with micro metastases in the SLN, recommended routine dissection for patients with metastases or micro metastases (>0.2 ≤ 2 mm) found on SNB, regardless of the method of detection [20], Many other studies, after adequate testing of the MSKCC nomogram, still share the ASCO approach and recommend routine AND [29,36-39].

Various other scoring systems have been proposed to simplify and improve the predictivity of this procedure. All these tools have been compared with the MSKCC nomogram. Among these, the Turkish score [30], the Cambridge nomogram [31], the Mayo nomogram [40], the Tenon score [41] and the Stanford online calculator [22].

The Stanford nomogram is the result of a multi-institutional study on a large sample set evaluated as univariate predictors of NSLN status tumor size (in cm and by AJCC T-size classification), tumor grade, hormone receptor status (ER and PR), angiolymphatic invasion (ALVI), size of SLN metastasis, and whether nodal tumor involvement is identified by hematoxylin and eosin stain. Age, though taken into account as a variable, was not significative, permitting to apply the results of the study even to older patients. Among the variables, the size of SN metastases and ALVI have shown to be the most effective. The Authors compared their results with the MSKCC nomogram, finding a higher accuracy with less need for information [22]. The Stanford model seems to perform well in some population, even better than the MSKCC one [39], but this conclusion has not be shared by other studies [29].

The Tenon score takes into account the presence of macro metastases in the SLN (yes = 2, no = 0), the histological tumor size in mm (>20 = 3, 11-20 = 1.5, <11 = 0) and the ratio between positive and total SLNs (1 = 2, 0.5-1 = 1, <0.5 = 0). The cut off value, over which is presumable the presence of additional metastases, is 3.5 [41,42]. The information useful to calculate the score can be obtained from frozen section, allowing the surgeon to decide during the surgery about the need to perform an axillary node dissection [42]. This characteristic can be even more useful in elderly patients. Tenon scoring system has performed, in some experience, better than MSKCC nomogram [35]. Unfortunately its good performance has not been truly validated yet, in fact, Swedish researchers observed a 14.1% rate of false negatives [43], while Unal and coll., on a small sample, observed a lowering in efficacy after neoadjuvant chemotherapy [44].

Despite the risk of leaving metastatic non sentinel node in the axilla, Ponzone et al [31] have stressed, in their appraisal of MSKCC nomogram, the importance of a tool able to select patient with low risk of axillary additional metastases and high surgical risk, who might not need a complete AND. A low risk of involvement of NSN in elderly should induce to compare life expectancy, surgical risk and quality of life, sparing unnecessary axillary dissection [32].

Nevertheless some Authors feel that when the sentinel node is negative this low invasive surgery should be avoided in elderly women.

Chagpar has so investigated this possibility; her study on 700 women, 70 years or older, with hormone-receptor positive BC, has showed that patients’ age, tumor size, and lymph vascular invasion can help to predict which women can be safely spared axillary node biopsy [45].

Therefore, researchers from the National Cancer Institute of Milan, Italy, performed a retrospective study on 70 years and older women with early stage BC and clinically negative axillary nodes undergone to conservative surgery. Their results showed that sparing AND was followed at 5 and 10 years by a 4.4% and 5.9% of axillary recurrence, respectively. In T1 cancers the rates of axillary node metastases were 3.1% and 4.1%, respectively. These data did not affect survival nor distant metastases; incidence and the Authors concluded that, in selected patients, axillary surgery should be avoided and performed at axillary recurrence [17].

However, before judging a woman too old for axillary surgery, let’s think about old ladies surviving more than 10 years to a conservative surgery without axillary dissection and then be devastated by a rapidly worsening monstrous lymph edema sustained by axillary recurrence, without a chance of relief.

Eventually, when AND is performed and metastases are found even in NSN, the prognostic value is well known and pN is already classified as pN1, pN2 or pN3 depending on the number of nodes involved (1-3; 4-9; 10 or more). Provided that a correct evaluation of axillary node staging needs at least 10 nodes to be harvested [46-48], the classification does not consider the total number of nodes collected.

But the predictive value of the total number of nodes has been shown by Somner and Coll in a study on 609 patients. The Authors found a higher rate of nodal metastases in patient in whom 16-20 nodes were collected (68%) respect to the ones with 1-15 (58%). So, the predictivity of finding negative nodes could be biased by the number of nodes retrieved. The result of the study, however, should take into account that nodal dissection was not performed on every BC patient but with a selective criteria including younger age, tumor size >2 cm, G III and/or lymph vascular invasion at core biopsy, and clinical positivity of axillary nodes. Therefore, in smaller and less aggressive tumors in older women the extension of dissection could be irrelevant on predictivity [49].

Ahn and coll., have evaluated the possibility of improving the accuracy of N staging by including the LN ratio, namely the ratio between the number of positive nodes and total number of nodes removed. An analysis on 15488 N+ BC conducted in Korea has permitted to classify patients into low- (≤0.20), intermediate- (>0.20 and ≤0.65), and high-risk (>0.65) LNR groups. The value of this further classifications is stronger in high risk patients as younger women, and c-Erb-B2 positive or triple negative tumours [50].

The importance of other prognostic factors, that in older women could replace axillary dissection or even SNB, will be assessed in following paragraphs.

### Tumor size

Although the size of tumor is a fundamental part of staging (T), and the first that can be assessed, only few studies describe it with full details in elderly women.

The largest study on tumor size is by Schomberg and colleagues [51], based on SEER database including 49.616 women aged 67 years or older. In this series, women aged 67 to 74 years showed the same rate of T1 neoplasms (34% of cases under 1 cm and 38-39% between 1 and 2 cm of size). With the increasing of age also the tumor size at diagnosis grew over 1 cm and far more of T2 (Table 3).

**Table 3:** Tumor size and age.

Tumor size\Age	67-69	70-74	75-79	80-84	85-89	>90
≤ 1 cm	34.7	34.2	32.4	28.4	21.5	12.5
1 to ≤2 cm	38.5	39.3	39.5	39.1	38.2	35.0
2 to ≤5 cm	22.2	21.8	23.8	27.8	34.4	44.9
>5 cm	0.9	0.9	1.1	1.1	1.8	3.2
unknown	3.7	3.9	3.3	3.5	4.0	4.5

Adapted From Schonberg et al, Journal of Clinical Oncology, 2010.

In other studies the stage distribution in elderly women varies largely with a 12% to 50% of tumor diagnosed in Stage IIIA or IIIB [14,52].

The limited significance of tumor size is stressed by Chagpar. She observed that in early stages, tumor size only permits to predict, together with other factors and with a huge variability, the probability of neoplastic involvement of axillary nodes [45].

### Lymphatic/vascular invasion

The value of lymph vascular invasion (LVI) in elderly women is discussed.

Chagpar has observed that the presence of LVI is an important predictor of axillary node involvement [45]. Other authors find the efficacy of this factor is higher in patients under sixties, together with tumor size (>1 cm) and mammographic pattern (category 5 with low density breast). In these cases LVI can predict, with a 95% of reliability, the presence of axillary node metastases [53].

In a study on more than 15000 patient with breast cancer [54], authors found that LVI was not an independent high-risk criterion. In fact, while in the high risk group, individuated by positive lymph nodes, tumor size >2 cm, high grade, hormone receptor-negative tumor, or age <35, the 5-year survival rate was significantly affected by the presence of LVI (65% and 85% in patient with or without LVI, respectively), in the low risk group survival was 98% and 94%, respectively [54].

It is possible, then, that LVI should be considered only as part of a panel of indicators, like in MSKCC nomogram, and should not be given *a per se *value [55].

### Endocrine receptors

Endocrine receptors, particularly estrogen receptors (ERs), are more frequent in older women. In the series by Gennari and Coll., among eldest women (≥75 years), 81% presented with ER+ while 61% was progesterone receptor (PR)+, compared to 78% and 52%, respectively, in the young postmenopausal group (50-64 yrs age) [56].

A more recent study by Chatzidaki has shown a just slightly lower frequency of positivity in a group of 137 women aged 80 years and older (ER+ 72% and PR+ 56%). This characteristic can offer a better chance of survival even in unfit women in whom surgery or chemotherapies are unsuitable [8].

A study on 82 women, median age 81 (range 62-93 yrs) and ER+ cancers, conducted by Osborn et al, showed optimistic results about the chance to treat these patients with primary endocrine therapy so that they can die with the cancer but not because of the cancer. Nevertheless in this series only 6 patients (7%) had a chance of a 10-years survival greater than 50%, while 23 (27%) have died between 1 and 77 months (mean 10.5 months), and 12 (15%) experienced progression while taking the therapy [57].

### HER-2Neu

The significance of Her-2Neu as indicative of a poorer prognosis is well known today. The availability of specific antibodies makes the fate of the women with HER+++ cancers slightly less unfavorable. The value of this marker is high even in older women. In a study on 153 women aged 70 years and older with stage I or II BC, Poltinnikov found Her-2Neu in 22% of patients and associating it with other unfavorable factors such as high histologic grade, T2 stage, positive axillary nodes, and with a global poorer outcome. Particularly affected were the rate of nodal and distant metastases (on 5-year follow-up, 70% for HErNeu+ versus 97% for HerNeu-negative) and the cause-specific 5-years survival (86% vs. 98%, respectively) [58]. Similar results were found by Durbecq and coll. who reported 19% of women 70 years and older with "luminal-B" tumors associated with high proliferation, high grade, large size and nodal invasion [59].

This is far more evident in older patients with interval cancer showing an higher frequency of ER-negative, PR-negative, or triple negative histotypes [60].

Thus, incidence of less favorable cancers like triple negative can vary with ethnic group but, in the same group the incidence is independent from age: triple-negative BCs are more frequent in black women regardless of age or body mass index [60].

### Specific markers able to evaluate response to therapies

Neopterin is a pteridin catabolic product of guanosine triphosphate (GTP), a purine nucleotide. It is synthesized by macrophages upon stimulation with the cytokine interferon-gamma and is indicative of a pro-inflammatory immune status so that it can be a marker of cellular immune system activation. Neopterin has been known for long time as a marker of immunological distress, linked to viral infections, like cytomegalovirus, or cancer. Its relationship with breast cancer has been evidenced principally for metastatic cancer [61].

Urinary neopterin has shown increased in about 20% of breast cancer patients, and a recent study has revealed its increase to be linked to age, 70 years or older, and to comorbidities (i.e. diabetes mellitus, atherosclerosis, hyperlipidemia, thyroid or cardiac disorders). Two or more comorbidities had a cumulative effect and were associated with higher levels of neopterin [62].

### Comorbidities and socio-economic factors

Many Authors have investigated the link between some specific disease and the prognosis of BC. Schrauder has observed that type 2 diabetes is often associated with more advanced cancer in older women but, after age and stage adjusted stratification, prognosis was similar in patient with or without diabetes. Though, he also observed that in patients with estrogens negative cancer, diabetes is associated with a more than doubled risk for distant metastases and an almost halved 5-years survival [63].

In contrast, obesity, often inquired as responsible for an higher frequency of breast cancer, has not shown any significant influence on prognosis [64], although previous study by Daling and coll. suggested that women in the highest quartile of BMI were 2.5 times more likely to die of their disease within 5 years of BC diagnosis compared with women in the lowest quartile [65].

A more recent study has demonstrated that even hypertension can be a prognostic factor, worsening the prognosis in elderly women with metastases from BC compared with younger [66].

But, more than a specific disease, it seems worth a comprehensive assessment of the health status of older patients with cancer. The evaluation of a geriatric patient, not differently from that of an oncologic patient, requires the assessment of the ability to perform the usual activity and is based on measures of the performance status, as the Karnofsky index or the ECOG (Eastern Cooperative oncology group) index. More specific indexes explore the ability to perform daily activities (ADL) like bathing or getting dress by themselves, or instrumental daily activities (IADL) as the use of a telephone or managing money [67].

It is over a decade that survival in geriatric oncologic patients has been evaluated in the light of a geriatric assessment, trying to introduce in the clinical practice a scoring system that could help to evaluate prognosis and to choose more appropriate cares [68,69].

In a recent report on 131 patients aged ≤75 years undergone radiotherapy after conservative surgery, comorbidities showed to be of great value in determining the prognosis. Patients with no or mild co-morbidities showed a significantly better survival while increasing severity of co-morbidity may sufficiently shorten remaining life expectancy to cancel gains with adjuvant radiotherapy [70].

This approach cannot help but could include the risk factor represented by comorbidities.

In 1994 Charlson and colleague proposed a simple method to classify the surgical risk from comorbidities showing, from a sample of 225 geriatric surgical patients, that each comorbidity had a relative risk of death of 1.4, about equivalent to being a decade older [71].

In the same year Satariano related the worsening of prognosis, for elderly BC patients, with the increasing number of morbidities affecting the survival or *per se *or modifying the therapeutic approach [72].

A recent large study has been conducted by Patnaik and Coll. on more than 64000 women with BC from SEER database, aimed to elucidate the influence of comorbidity on survival [73]. They found 13 morbid conditions (previous cancer, myocardial infarction, congestive heart failure, peripheral vascular diseases, stroke, chronic obstructive pulmonary disease, dementia, paralysis, diabetes, chronic renal failure, liver disease, ulcers, rheumatoid arthritis) whose presence as single factor worsens the overall survival acting as a stage shift. Women with stage I tumor and one of these factors had a life expectancy similar or poorer than those with stage II cancer. To exclude the impact of growing age, the comparison has been made for class of age: 65-74, 75-84 and 85 and older. In all classes it has been observed the same effect of comorbidity [73].

A combined age-comorbidity score has been considered an useful tool for estimating risk in geriatric patients, giving the basis for further evaluations. The use of a comprehensive geriatric assessment (CGA) is part of this effort aimed to integrate information about not only the cancer and the general health status but also to psycho-social element affecting the chance of the patient to interact with physician and caregivers and to accomplish the routine of the oncological therapies [74]. Experience even on other types of cancer have showed that a wide use of CGA-driven treatments may result in better cure rates, both in fit and unfit patients [75].

A complex system of assessment has been proposed by Clough-Gorr and coll., analyzing a sample of 660 patients with BC followed for 10 years. They studied 4 classes of factors, defined domains. For the demographic class they included just the financial status (having or not having adequate finances to meet needs); the Health status was assessed by using the Charlson comorbidity index (CCI) scaled from 0 to 3, with higher scores indicating more comorbidity, and Body mass index (≤30 kg/m^2 ^versus obesity (>30 kg/m^2^); the functional class was expressed by the number of limiting physical functions (none or ≥1 limitation); and the forth class included psychosocial factors evaluated by general mental health (by Mental Health Index -MHI5- a five-item measure of mental health from the Medical Outcomes Study Short Form scale from 0 to 100 with a score of ≥80 considered as good general mental health) and social support, measured using eight items from the 19-item Medical Outcomes Study Social Support Scale (MOS-SSS) including emotional and instrumental social support items. They observed that deficit in three or more domains was associated with and higher mortality [69].

The impact of socio-economic status as well was elucidated in a study conducted on 1081 patients from the Liguria Region Cancer Registry patients with similar results to those observed by Clough-Gorr [76].

### Under treatment

Many evidences show that older cancer patients in good health can obtain, from adequate treatment, the same benefit than younger patients [68,77].

Livi et al, in a study on 15500 women over 65 found that the type of surgery, along with histotype, pN status and pT status were the only independent prognostic factors, while age was not, neither for disease specific survival, nor disease free survival. It is important to warrant older women the same treatments offered to younger women, unless unworthy for limited life-expectancy, to avoid that age becomes not only a risk factor for BC, but even a poor prognostic factor [78].

The study by Silliman on 1859 women 65 years and older, has found that under-treatment is a major risk factor for recurrence and death from BC [79].

Though, age is often seen as a reason for sparing aggressive treatments, either surgical or chemo- or radio-therapeutic or avoiding invasive follow-up. The reduced expectancy of life due to the age and to other diseases often induces both physicians and patients to choose less aggressive therapies, so older women undergo to not-standard treatments more often than the younger ones [20,78-81].

The rate of non conventional treatments is largely variable between 10% [82] and 39% in Malaysian series [6].

On the other side, age becomes often a reason to overlook the aesthetics and the psychophysical aspects. Wang, on a survey of 31298 patients with early BC from Australia and New Zealand between 1999 and 2006, observed that women older than 70 years were more likely to receive mastectomy in the place of breast conserving surgery or no surgery at all (3.5%) than younger counterpart [83].

The impact of under-treatment is often questioned and is not reliably evaluable as older women are not usually included in clinical trials.

Some Authors affirm that in older women undergoing under-treatments by conventional criteria, the rates of local recurrence and distant metastasis are not increased in comparison with conventionally treated elderly patients [82]. In contrast, Yood et al in a cohort study on 1837 women aged 65 or older, treated for stage I or II breast cancer observed a significative difference between those treated with standard surgery (mastectomy (M) or breast conserving surgery (BCS) + RT) an those who received BCS alone. At 10 years follow up the risk of death for those undergone BCS alone was double than for those undergone standard surgery, even after adjustment for demographics and tumor characteristics [84].

A last factor can be seen in some delay in the diagnosis observed in older women. In multivariate analysis, increased time to surgery was associated with older age but this factor did not appear related with a poorer prognosis since modest time intervals from imaging to surgery are not significantly associated with change in tumor size [85].

Regarding the adjuvant therapies, use of tamoxifen in women with receptor-positive tumors is a relatively simple decision in light of its favorable toxicity profile [86], thus it has been proven to significantly reduce the chance of developing distant metastasis in node-negative elderly patients with invasive tumors [82]. Yood, in the cited paper, reports a significantly longer survival in patients assuming tamoxifen for 5 years or more than in those who had HT for 1 year or less [84].

Nevertheless, in the series by Owusu, in as many as 14% of women older than 65 years has been observed a change in score due to arising of pulmonary chronic diseases that have been associated with discontinuation of assumption of tamoxifen [88]. It has not been specified if the pulmonary complications were due to the therapy or just therapy-associated.

Muss observed that, although older women can expect from endocrine therapies the same improvement in survival than younger women, when HR positive, the therapy-related mortality is higher [89].

Delivery of adjuvant chemotherapy is instead a more complicated decision [90]. Paik observed that only patients with high-risk of developing metastases within 10 years have benefitted from chemotherapy administration [91]. Some author propose that the patients’ wishes, estimated life expectancy, presence of comorbid conditions, and estimated benefit from treatment should be considered before any kind of adjuvant therapy [86,87].

However, it is true that many 80 years or older patients receive less than standard surgical treatment [77,78], or chemotherapy [92]. Other elderly patients show poor compliance or still are unable to correctly assume medications and this can determinate an higher risk of side effects, even fatal [68].

## Conclusions

Breast cancer in elderly is not definitely a less aggressive disease compared with the cancer arising in younger women. Predictive factors are the same and must be assessed with the same attention reserved to younger women although many patients should be considered for less invasive treatments. Socioeconomic factors and the general health status are thus effective factors affecting prognosis, modifying the life expectancy and the compliance to the therapies.

## Competing interests

The authors declare that they have no competing interests.

## Authors' contributions

A.C.: conception and design, interpetration of data, given final approval of the version to be published. M.DV.: conception and design, acquisition of data, interpretation of data, drafting the manuscript, given final approval of the version to be published. AZ.: acquisition of data, drafting the manuscript, given final approval of the version to be published. An.Cav.: acquisition of data, given final approval of the version to be published. G.P.: acquisition of data, drafting the manuscript, given final approval of the version to be published. M.M.: conception and design, critical revision, given final approval of the version to be published. G.B.: acquisition of data, drafting the manuscript, given final approval of the version to be published. M.B.: conception and design, critical revision, given final approval of the version to be published.

## Authors' information

AC: Full Professor of Surgery at University of Catania, School of Medicine. MDV: Researcher at Dept of Surgery, University of Catania, School of Medicine. AZ: Associate Professor of Surgery, University of Catania, School of Medicine. AnCav: Surgeon at University of Catania, School of Medicine, Catania University Hospital. GP: Resident in Surgery at University of Catania, School of Medicine. MM: Chief of Department of Radiology, Mediterranean Cancer Institute. GB: Resident in Surgery; University of Catania, School of Medicine. MB: MD, PhD, Oncologist at CRO IRCCS, Aviano.

## Supplementary Material

Additional file 1 **Principal studies correlating older ages and prognostic factors**. These studies fulfilled the criteria for inclusion Click here for file

Additional file 2 **Other studies consulted and cited even not fulfilling criteria**. The studies listed in this section were relevant, though not fulfilling criteria (i.e.: consensus conference, validation study, studies including all ages patients or not aimed to survival.). The aim of the study is descripted too. Click here for file

## Declarations

Funding for publication of this article has been granted to Dr Di Vita by the University of Catania.

This article has been published as part of *BMC Surgery *Volume 13 Supplement 2, 2013: Proceedings from the 26th National Congress of the Italian Society of Geriatric Surgery. The full contents of the supplement are available online at http://www.biomedcentral.com/bmcsurg/supplements/13/S2

## References

[B1] Howlader N Noone AM Krapcho M Neyman N Aminou R Altekruse SF Kosary CL Ruhl J Tatalovich Z Cho H Mariotto A Eisner MP Lewis DR Chen H-S Feuer EJ Cronin K-A **SEER Cancer Statistics Review, 1975-2009 (Vintage 2009 Populations)** National Cancer Institute Bethesda, MD

[B2] Ali AM Greenberg D Wishart GC Pharoah P Patient and tumour characteristics, management, and age-specific survival in women with breast cancer in the East of England Br J Cancer 2011 104 564 570 21326244 10.1038/bjc.2011.14 PMC3049594

[B3] Lavelle K Todd C Moran A Howell A Bundred N Campbell M Non-standard management of breast cancer increases with age in the UK: a population based cohort of women > or =65 years Br J Cancer 2007 96 1197 1203 17387342 10.1038/sj.bjc.6603709 PMC2360138

[B4] Associazione Italiana Registri Tumori, Epidemiologia & Prevenzione 2006 30 suppl 2

[B5] Wyld L Garg DK Kumar ID Brown H Reed MW Stage and treatment variation with age in postmenopausal women with breast cancer: compliance with guidelines Br J Cancer 2004 90 1486 1491 15083173 10.1038/sj.bjc.6601742 PMC2409727

[B6] Phua CE Bustam AZ Yip CH Taib NA Prognostic factors for elderly breast cancer patients in University Malaya Medical Centre, Malaysia Asian Pac J Cancer Prev 2010 11 1205 1211 21198264

[B7] Williams BA Lindquist K Sudore RL Covinsky KE Walter LC Screening mammography in older women. Effect of wealth and prognosis Arch Intern Med 2008 168 514 520 18332298 10.1001/archinternmed.2007.103

[B8] Chatzidaki P Mellos C Briese V Mylonas I Does primary breast cancer in older women (≥80 years) have unfavorable histological characteristics? Arch Gynecol Obstet 2011 284 705 712 20949358 10.1007/s00404-010-1697-5

[B9] Serra R Buffone G Perri P Renne M Amato B de Franciscis S Male breast cancer manifesting as Cephalic Vein Thrombosis in a 70-year-old patient Ann Vasc Surg 2013 in press 10.1016/j.avsg.2013.01.016 23988541

[B10] van de Water W Markopoulos C van de Velde CJ Seynaeve C Hasenburg A Rea D Putter H Nortier JW de Craen AJ Hille ET Bastiaannet E Hadji P Westendorp RG Liefers GJ Jones SE Association between age at diagnosis and disease-specific mortality among postmenopausal women with hormone receptor-positive breast cancer JAMA 2012 8 307 590 597 22318280 10.1001/jama.2012.84

[B11] Ramirez AJ Westcombe AM Burgess CC Sutton S Littlejohns P Richards MA Factors predicting delayed presentation of symptomatic breast cancer: a systematic review Lancet 1999 353 1127 1131 10209975 10.1016/s0140-6736(99)02142-x

[B12] Richards MA Smith P Ramirez AJ Fentiman IS Rubens RD The influence on survival of delay in the presentation and treatment of symptomatic breast cancer Br Journal of Cancer 1999 79 858 864 10.1038/sj.bjc.6690137 PMC2362673 10070881

[B13] Leonard R Ballinger R Cameron D Ellis P Fallowfield L Gosney M Johnson L Kilburn LS Makris A Mansi J Reed M Ring A Robinson A Simmonds P Thomas G Bliss JM Adjuvant chemotherapy in older women (ACTION) study - what did we learn from the pilot phase? Br J Cancer 2011 105 1260 1266 21989185 10.1038/bjc.2011.377 PMC3241551

[B14] Solej M Sanguinett A Ragusa M de Falco M Sperlongano P Calzolari F Parmeggiani D Misso C Piatto A Parmeggiani U Avenia N Locally advanced breast cancer in elderly patients: treatment standardised or tailored to individual needs? Chirurgia Italiana 2007 59 829 833 18360988

[B15] Di Vita M Berretta M Zanghi A Cacopardo B Cavallaro A Lombardi D Lo Menzo E Cappellani A Bioclinical markers in breast cancer: updates and perspectives Front Biosci (Schol Ed) 2010 2 343 358 20036952 10.2741/s69

[B16] Martelli G Boracchi P de Palo M Pilotti S Oriana S Zucali R Daidone MG De Palo G A randomized trial comparing axillary dissection to no axillary dissection in older patients with T1N0 breast cancer: results after 5 years of follow-up Ann Surg 2005 242 1 6 discussion 7-9 15973094 10.1097/01.sla.0000167759.15670.14 PMC1357697

[B17] Martelli G Miceli R Daidone MG Vetrella G Cerrotta AM Piromalli D Agresti R Axillary dissection versus no axillary dissection in elderly patients with breast cancer and no palpable axillary nodes: results after 15 years of follow-up Ann Surg Oncol 2011 18 125 133 20652755 10.1245/s10434-010-1217-7 PMC3018257

[B18] Albrand G Terret C Early breast cancer in the elderly: assessment and management considerations Drugs Aging 2008 25 35 45 18184027 10.2165/00002512-200825010-00004

[B19] Audisio RA Bozzetti F Gennari R Jaklitsch MT Koperna T Longo WE Wiggers T Zbar AP The surgical management of elderly cancer patients; recommendations of the SIOG surgical task force Eur J Cancer 2004 40 926 938 15093567 10.1016/j.ejca.2004.01.016

[B20] Hieken TJ Nettnin S Velasco JM The value of sentinel lymph node biopsy in elderly breast cancer patients Am J Surg 2004 188 440 442 15474445 10.1016/j.amjsurg.2004.06.028

[B21] Gennari R Curigliano G Rotmensz N Robertson C Colleoni M Zurrida S Nol F de Braud F Orlando L Leonardi MC Galimberti V Intra M Veronesi P Renne G Cinieri S Audisio RA Luini A Orecchia R Viale G Goldhirsch A Breast carcinoma in elderly women Cancer 2004 101 1302 1310 15316944 10.1002/cncr.20535

[B22] Kohrt HE Olshen RA Bermas HR Goodson WH Wood DJ Henry S Rouse RV Bailey L Philben VJ Dirbas FM Dunn JJ Johnson DL Wapnir IL Carlson RW Stockdale FE Hansen NM Jeffrey SS New models and online calculator for predicting non-sentinel lymph node status in sentinel lymph node positive breast cancer patients BMC Cancer 2008 8 66 Bay Area SLN Study 18315887 10.1186/1471-2407-8-66 PMC2311316

[B23] Biganzoli L Wildiers H Oakman C Marotti L Loibl S Kunkler I Reed M Ciatto S Voogd AC Brain E Cutuli B Terret C Gosney M Aapro M Audisio R Management of elderly patients with breast cancer: updated recommendations of the International Society of Geriatric Oncology (SIOG) and European Society of Breast Cancer Specialists (EUSOMA) Lancet Oncol 2012 13 4 e148 e160 Apr 22469125 10.1016/S1470-2045(11)70383-7

[B24] Wildiers H Kunkler I Biganzoli L Fracheboud J Vlastos G Bernard-Marty C Hurria A Extermann M Girre V Brain E Audisio RA Bartelink H Barton M Giordano SH Muss H Aapro M Management of breast cancer in elderly individuals: recommendations of the International Society of Geriatric Oncology Lancet Oncol 2007 8 12 1101 1115 International Society of Geriatric Oncology 18054880 10.1016/S1470-2045(07)70378-9

[B25] Lyman GH Giuliano AE Somerfield MR Benson AB III Bodurka DC Burstein HJ Cochran AJ Cody HS III Edge SB Galper S Hayman JA Kim TY Perkins CL Podoloff DA Sivasubramaniam VH Turner RR Wahl R Weaver DL Wolff AC Winer EP American Society of Clinical Oncology guideline recommendations for sentinel lymph node biopsy in early-stage breast cancer J Clin Oncol 2005 23 7703 7720 American Society of Clinical Oncology 16157938 10.1200/JCO.2005.08.001

[B26] Chu KU Turner RR Hansen NM Brennan MB Bilchik A Giuliano AE Do all patients with sentinel node metastasis from breast carcinoma need complete axillary node dissection? Ann Surg 1999 229 536 541 10203087 10.1097/00000658-199904000-00013 PMC1191740

[B27] Van Zee KJ Manasseh DM Bevilacqua JL Boolbol SK Fey JV Tan LK Borgen PI Cody HS III Kattan MW A nomogram for predicting the likelihood of additional nodal metastases in breast cancer patients with a positive sentinel node biopsy Ann Surg Oncol 2003 10 1140 1151 14654469 10.1245/aso.2003.03.015

[B28] Smidt ML Kuster DM van der Wilt GJ Thunnissen FB Van Zee KJ Strobbe LJ Can the Memorial Sloan-Kettering Cancer Center nomogram predict the likelihood of nonsentinel lymph node metastases in breast cancer patients in the Netherlands? Ann Surg Oncol 2005 12 1066 1072 16244802 10.1245/ASO.2005.07.022

[B29] Orsoni M Clerc J Golfier F Cortet M Raudrant D Krauth JS Axillary lymph node dissection in the case of sentinel lymph node micrometastatic invasion: evaluation of three predictive models Eur J Obstet Gynecol Reprod Biol 2011 158 334 337 21669485 10.1016/j.ejogrb.2011.05.020

[B30] Gur AS Unal B Ozbek U Ozmen V Aydogan F Gokgoz S Gulluoglu BM Aksaz E Ozbas S Baskan S Koyuncu A Soran A Validation of breast cancer nomograms for predicting the non-sentinel lymph node metastases after a positive sentinel lymph node biopsy in a multi-center study Eur J Surg Oncol 2010 36 30 35 Turkish Federation of Breast Disease Associations Protocol MF08-01 investigators. 19535217 10.1016/j.ejso.2009.05.007

[B31] Pal A Provenzano E Duffy SW Pinder SE Purushotham AD A model for predicting non-sentinel lymph node metastatic disease when the sentinel lymph node is positive Br J Surg 2008 95 302 309 17876750 10.1002/bjs.5943

[B32] Ponzone R Maggiorotto F Mariani L Jacomuzzi ME Magistris A Mininanni P Biglia N Sismondi P Comparison of two models for the prediction of nonsentinel node metastases in breast cancer Am J Surg 2007 193 686 692 17512277 10.1016/j.amjsurg.2006.09.031

[B33] Alran S de Rycke Y Fourchotte V Charitansky H Laki F Falcou MC Benamor M Freneaux P Salmon RJ Sigal-Zafrani B Validation and limitations of use of a breast cancer nomogram predicting the likelihood of non-sentinel node involvement after positive sentinel node biopsy Ann Surg Oncol 2007 14 2195 2201 Institut Curie Breast Cancer Study Group 17294071 10.1245/s10434-006-9331-2

[B34] Sasada T Murakami S Kataoka T Ohara M Ozaki S Okada M Ohdan H Nomogram to predict the risk of non-sentinel lymph node metastasis in Japanese breast cancer patients Surg Today 2012 42 245 249 Memorial Sloan-Kettering Cancer Center 22167482 10.1007/s00595-011-0088-2

[B35] Moghaddam Y Falzon M Fulford L Williams NR Keshtgar MR Comparison of three mathematical models for predicting the risk of additional axillary nodal metastases after positive sentinel lymph node biopsy in early breast cancer Br J Surg 2010 97 1646 1652 20641049 10.1002/bjs.7181

[B36] D’Eredità G Troilo VL Giardina C Napoli A Rubini G Fischetti F Berardi T Sentinel lymph node micrometastasis and risk of non-sentinel lymph node metastasis: validation of two breast cancer nomograms Clin Breast Cancer 2010 10 445 451 21147687 10.3816/CBC.2010.n.058

[B37] van den Hoven I Kuijt GP Voogd AC van Beek MW Roumen RM Value of Memorial Sloan-Kettering Cancer Center nomogram in clinical decision making for sentinel lymph node-positive breast cancer Br J Surg 2010 97 1653 1658 20641050 10.1002/bjs.7186

[B38] Klar M Jochmann A Foeldi M Stumpf M Gitsch G Stickeler E Watermann D The MSKCC nomogram for prediction the likelihood of non-sentinel node involvement in a German breast cancer population Breast Cancer Res Treat 2008 112 523 531 18172758 10.1007/s10549-007-9884-1

[B39] Hidar S Harrabi I Benregaya L Fatnassi R Khelifi A Benabdelkader A Trabelsi A Bouaouina N Ben Ahmed S Bib M Khaïri H Validation of nomograms to predict the risk of non-sentinels lymph node metastases in North African Tunisian breast cancer patients with sentinel node involvement Breast 2011 20 26 30 20729084 10.1016/j.breast.2010.07.006

[B40] Mittendorf EA Hunt KK Boughey JC Bassett R Degnim AC Harrell R Yi M Meric-Bernstam F Ross MI Babiera GV Kuerer HM Hwang RF Incorporation of sentinel lymph node metastasis size into a nomogram predicting nonsentinel lymph node involvement in breast cancer patients with a positive sentinel lymph node Ann Surg Oncol 2012 255 109 115 10.1097/SLA.0b013e318238f461 PMC4760742 22167004

[B41] Barranger E Coutant C Flahault A Delpech Y Darai E Uzan S An axilla scoring system to predict non-sentinel lymph node status in breast cancer patients with sentinel lymph node involvement Breast Cancer Res Treat 2005 91 113 119 15868438 10.1007/s10549-004-5781-z

[B42] Coutant C Rouzier R Fondrinier E Marchal F Guillemin F Seince N Rodrigues A Darai E Uzan S Barranger E Validation of the Tenon breast cancer score for predicting non-sentinel lymph node status in breast cancer patients with sentinel lymph node metastasis: a prospective multicenter study Breast Cancer Res Treat 2009 113 537 543 18340527 10.1007/s10549-008-9967-7

[B43] Andersson Y Frisell J de Boniface J Bergkvist L Prediction of non-sentinel lymph node status in breast cancer patients with sentinel lymph node metastases: evaluation of the tenon score Breast Cancer (Auck) 2012 6 31 38 10.4137/BCBCR.S8642 PMC3273320 22346360

[B44] Unal B Gur AS Ahrendt G Johnson R Bonaventura M Soran A Can nomograms predict non-sentinel lymph node metastasis after neoadjuvant chemotherapy in sentinel lymph node-positive breast cancer patients? Clin Breast Cancer 2009 9 92 95 19433389 10.3816/CBC.2009.n.017

[B45] Chagpar AB McMasters KM Edwards MJ Can sentinel node biopsy be avoided in some elderly breast cancer patients? Ann Surg 2009 249 455 460 North American Fareston Tamoxifen Adjuvant Trial 19247034 10.1097/SLA.0b013e318194d16b

[B46] Chagpar AB Scoggins CR Martin RC II Sahoo S Carlson DJ Laidley AL El-Eid SE McGlothin TQ McMasters KM Factors determining adequacy of axillary node dissection in breast cancer patients Breast J 2007 13 233 237 University of Louisville Breast Sentinel Lymph Node Study 17461896 10.1111/j.1524-4741.2007.00415.x

[B47] Carter CL Allen C Henson DE Relation of tumor size, lymph node status, and survival in 24,740 breast cancer cases Cancer 1989 63 181 187 2910416 10.1002/1097-0142(19890101)63:1<181::aid-cncr2820630129>3.0.co;2-h

[B48] Fitzgibbons PL Page DL Weaver D Thor AD Allred DC Clark GM Ruby SG O’Malley F Simpson JF Connolly JL Hayes DF Edge SB Lichter A Schnitt SJ Prognostic factors in breast cancer College of American Pathologists Consensus Statement 1999 Arch Pathol Lab Med 2000 124 966 978 10888772 10.5858/2000-124-0966-PFIBC

[B49] Somner JE Dixon JM Thomas JS Node retrieval in axillary lymph node dissections: recommendations for minimum numbers to be confident about node negative status J Clin Pathol 2004 57 845 848 15280406 10.1136/jcp.2003.015560 PMC1770384

[B50] Ahn SH Kim HJ Lee JW Gong GY Noh DY Yang JH Jung SS Park HY Lymph node ratio and pN staging in patients with node-positive breast cancer: a report from the Korean breast cancer society Breast Cancer Res Treat 2011 130 507 515 21858659 10.1007/s10549-011-1730-9

[B51] Amato B Rispoli C Iannone L Testa S Compagna R Rocco N Surgical margins of resection for breast cancer: Current evidence Minerva Chirurgica 2012 67 5 445 452 23232484

[B52] Solej M Ferronato M Nano M Locally advanced breast cancer in the elderly: curettage mastectomy Tumori 2005 91 321 324 16277097 10.1177/030089160509100407

[B53] Wasuthit Y Kongdan Y Suvikapakornkul R Lertsithichai P Chirappapha P Predictive factors of axillary lymph node metastasis in breast cancer J Med Assoc Thai 2011 94 65 70 21425730

[B54] Ejlertsen B Jensen MB Rank F Rasmussen BB Christiansen P Kroman N Kvistgaard ME Overgaard M Toftdahl DB Mouridsen HT Population-based study of peritumoral lymphovascular invasion and outcome among patients with operable breast cancer J Natl Cancer Inst 2009 101 729 735 Danish Breast Cancer Cooperative Group 19436035 10.1093/jnci/djp090

[B55] Ramjeesingh R Quan ML Gardner S Holloway CM Prediction of involvement of sentinel and nonsentinel lymph nodes in a Canadian population with breast cancer Can J Surg 2009 52 23 30 19234648 PMC2637641

[B56] Gennari R Rotmensz N Perego E dos Santos G Veronesi U Sentinel node biopsy in elderly breast cancer patients Surg Oncol 2004 13 193 196 15615656 10.1016/j.suronc.2004.08.011

[B57] Osborn G Jones M Champ C Gower-Thomas K Vaughan-Williams E Is primary endocrine therapy effective in treating the elderly, unfit patient with breast cancer? Ann R Coll Surg Engl 2011 93 286 289 21944793 10.1308/003588411X571917 PMC3363077

[B58] Poltinnikov IM Rudoler SB Tymofyeyev Y Kennedy J Anne PR Curran WJ Jr Impact of Her-2 Neu overexpression on outcome of elderly women treated with wide local excision and breast irradiation for early stage breast cancer: an exploratory analysis Am J Clin Oncol 2006 29 71 79 16462507 10.1097/01.coc.0000197696.48980.11

[B59] Durbecq V Ameye L Veys I Paesmans M Desmedt C Sirtaine N Sotiriou C Bernard-Marty C Nogaret JM Piccart M Larsimont D A significant proportion of elderly patients develop hormone-dependant "luminal-B" tumours associated with aggressive characteristics Crit Rev Oncol Hematol 2008 67 1 80 92 Jul 18313937 10.1016/j.critrevonc.2007.12.008

[B60] Lowery JT Byers T Kittelson J Hokanson JE Mouchawar J Lewin J Merrick D Hines L Singh M Differential expression of prognostic biomarkers between interval and screen-detected breast cancers: does age or family history matter? Breast Cancer Res Treat 2011 129 211 219 21431872 10.1007/s10549-011-1448-8 PMC4675131

[B61] Yildirim Y Gunel N Coskun U Pasaoglu H Aslan S Cetin A Serum neopterin levels in patients with breast cancer Med Oncol 2008 25 403 407 18320364 10.1007/s12032-008-9054-2

[B62] Melicharovà K Kalàbovà H Krcmovà L Urbànek L Solichovà D Melichar B Effect of comorbidity on urinary neopterin in patients with breast carcinoma Eur J Cancer Care (English Ed online) 2010 19 340 345 10.1111/j.1365-2354.2008.01058.x 19912307

[B63] Schrauder MG Fasching PA Häberle L Lux MP Rauh C Hein A Bayer CM Heusinger K Hartmann A Strehl JD Wachter DL Schulz-Wendtland R Adamietz B Beckmann MW Loehberg CR Diabetes and prognosis in a breast cancer cohort J Cancer Res Clin Oncol 2011 137 6 975 983 Jun 21132511 10.1007/s00432-010-0960-2 PMC11827943

[B64] Cappellani A Di Vita M Zanghi A Cavallaro A Piccolo G Veroux M Berretta M Malaguarnera M Canzonieri V Lo Menzo E Diet, obesity and breast cancer: an update Front Biosci (Schol Ed) 2012 4 90 108 Jan 1 22202045 10.2741/s253

[B65] Daling JR Malone KE Doody DR Johnson LG Gralow JR Porter PL Relation of body mass index to tumor markers and survival among young women with invasive ductal breast carcinoma Cancer 2001 92 4 720 729 11550140 10.1002/1097-0142(20010815)92:4<720::aid-cncr1375>3.0.co;2-t

[B66] Jung SY Rosenzweig M Linkov F Brufsky A Weissfeld JL Sereika SM Comorbidity as a mediator of survival disparity between younger and older women diagnosed with metastatic breast cancer Hypertension 2012 59 205 211 22184319 10.1161/HYPERTENSIONAHA.111.171736

[B67] Extermann M Hurria A Comprehensive geriatric assessment for older patients with cancer J Clin Oncol 2007 25 1824 1831 17488980 10.1200/JCO.2007.10.6559

[B68] Repetto L Fratino L Audisio RA Venturino A Gianni W Vercelli M Parodi S Dal Lago D Gioia F Monfardini S Aapro MS Serraino D Zagonel V Comprehensive geriatric assessment adds information to Eastern Cooperative Oncology Group performance status in elderly cancer patients: an Italian Group for Geriatric Oncology Study J Clin Oncol 2002 20 494 502 11786579 10.1200/JCO.2002.20.2.494

[B69] Clough-Gorr KM Stuck AE Thwin SS Silliman RA Older breast cancer survivors: geriatric assessment domains are associated with poor tolerance of treatment adverse effects and predict mortality over 7 years of follow-up J Clin Oncol 2010 28 380 386 20008637 10.1200/JCO.2009.23.5440 PMC2815700

[B70] Fiorica F Berretta M Ursino S Fisichella R Lleshi A Fiorica G Stefanelli A Zini G Tirelli U Zanghi A Cappellani A Berretta S Cartei F Adjuvant radiotherapy on older and oldest breast cancer patients after conservative surgery: A retrospective analysis Arch Gerontol Geriatr 2012 55 2 283 288 Sep 22037244 10.1016/j.archger.2011.10.002

[B71] Charlson M Szatrowski TP Peterson J Gold J Validation of a combined comorbidity index J Clin Epidemiol 1994 47 1245 1251 7722560 10.1016/0895-4356(94)90129-5

[B72] Satariano WA Ragland DR The effect of comorbidity on 3-year survival of women with primary breast cancer Ann Intern Med 1994 120 104 110 8256968 10.7326/0003-4819-120-2-199401150-00002

[B73] Patnaik JL Byers T Di Guiseppi C Denberg TD Dabelea D The influence of comorbidities on overall survival among older women diagnosed with breast cancer J Natl Cancer Inst 2011 103 1101 1111 21719777 10.1093/jnci/djr188 PMC3139585

[B74] Clough-Gorr KM Silliman RA Translation Requires Evidence: Does Cancer-Specific CGA Lead to Better Care and Outcomes? Oncology 2008 22 (Williston Park) 925 928 20798779 PMC2927829

[B75] Spina M Balzarotti M Uziel L Ferreri AJ Fratino L Magagnoli M Talamini R Giacalone A Ravaioli E Chimienti E Berretta M Lleshi A Santoro A Tirelli U Modulated chemotherapy according to modified comprehensive geriatric assessment in 100 consecutive elderly patients with diffuse large B-cell lymphoma Oncologist 2012 17 6 838 846 22610154 10.1634/theoncologist.2011-0417 PMC3380883

[B76] Quaglia A Lillini R Casella C Giachero G Izzotti A Vercelli M The combined effect of age and socio-economic status on breast cancer survival Crit Rev Oncol Hematol 2011 77 210 220 Liguria Region Tumour Registry 20227888 10.1016/j.critrevonc.2010.02.007

[B77] Crivellari D Spazzapan S Lombardi D Berretta M Magri MD Sorio R Scalone S Veronesi A Treatment of older breast cancer patients with high recurrence risk Crit Rev Oncol Hematol 2003 46 241 246 12791423 10.1016/s1040-8428(03)00023-4

[B78] Livi L Paiar F Saieva C Simontacchi G Nori J Sanchez L Santini R Mangoni M Fondelli S Distante V Bianchi S Biti G Breast cancer in the elderly: treatment of 1500 patients Breast J 2006 12 353 359 16848846 10.1111/j.1075-122X.2006.00275.x

[B79] Silliman RA When cancer in older adults is undermanaged: the breast cancer story J Am Geriatr Soc 2009 57 Suppl 2 S259 S261 20122024 10.1111/j.1532-5415.2009.02506.x PMC2908961

[B80] Bouchardy C Rapiti E Fioretta G Laissue P Neyroud-Caspar I Schäfer P Kurtz J Sappino AP Vlastos G Undertreatment strongly decreases prognosis of breast cancer in elderly women J Clin Oncol 2003 21 19 3580 3587 Oct 1 12913099 10.1200/JCO.2003.02.046

[B81] Janssen-Heijnen ML Maas HA Houterman S Lemmens VE Rutten HJ Coebergh JW Comorbidity in older surgical cancer patients: influence on patient care and outcome European Journal of Cancer 2007 43 2179 2193 17681780 10.1016/j.ejca.2007.06.008

[B82] Gajdos C Tartter PI Bleiweiss IJ Lopchinsky RA Bernstein JL The consequence of undertreating breast cancer in the elderly J Am Coll Surg 2001 192 698 707 11400963 10.1016/s1072-7515(01)00832-8

[B83] Wang J Kollias J Boult M Babidge W Zorbas HN Roder D Maddern G Patterns of surgical treatment for women with breast cancer in relation to age Breast J 2010 16 60 65 19889171 10.1111/j.1524-4741.2009.00828.x

[B84] Yood MU Owusu C Buist DS Geiger AM Field TS Thwin SS Lash TL Prout MN Wei F Quinn VP Frost FJ Silliman RA Mortality impact of less-than-standard therapy in older breast cancer patients J Am Coll Surg 2008 206 1 66 75 Jan 18155570 10.1016/j.jamcollsurg.2007.07.015

[B85] Wagner JL Warneke CL Mittendorf EA Bedrosian I Babiera GV Kuerer HM Hunt KK Yang W Sahin AA Meric-Bernstam F Delays in primary surgical treatment are not associated with significant tumor size progression in breast cancer patients Ann Surg 2011 254 119 124 21494124 10.1097/SLA.0b013e318217e97f PMC4345121

[B86] Kimmick GG Muss HB Systemic therapy for older women with breast cancer Oncology 2001 15 (Williston Park) 280 291 discussion 291-292, 295-296, 299 11301828

[B87] Berretta M Cappellani A Fiorica F Nasti G Frustaci S Fisichella R Bearz A Talamini R Lleshi A Tambaro R Cocciolo A Ristagno M Bolognese A Basile F Meneguzzo N Berretta S Tirelli U FOLFOX4 in the treatment of metastatic colorectal cancer in elderly patients: a prospective study Arch Gerontol Geriatr 2011 52 1 89 93 Jan-Feb 20211502 10.1016/j.archger.2010.02.006

[B88] Owusu C Buist DS Field TS Lash TL Thwin SS Geiger AM Quinn VP Frost F Prout M Yood MU Wei F Silliman RA Predictors of tamoxifen discontinuation among older women with estrogen receptor-positive breast cancer J Clin Oncol 2008 25 549 555 18071188 10.1200/JCO.2006.10.1022

[B89] Muss HB Woolf S Berry D Cirrincione C Weiss RB Budman D Wood WC Henderson IC Hudis C Winer E Cohen H Wheeler J Norton L Adjuvant chemotherapy in older and younger women with lymph node-positive breast cancer JAMA 2005 293 1073 1081 Cancer and Leukemia Group B 15741529 10.1001/jama.293.9.1073

[B90] Crivellari D Toffoli G Lombardi D Berretta M Sorio R Magri MD Spazzapan S Scuderi C Veronesi A Idarubicin Tumori 2002 88 1 Suppl 1 S73 S74 11989932 10.1177/030089160208800122

[B91] Paik S Shak S Tang G Kim C Baker J Cronin M Baehner FL Walker MG Watson D Park T Hiller W Fisher ER Wickerham DL Bryant J Wolmark N A multigene assay to predict recurrence of tamoxifen-treated, node-negative breast cancer N Engl J Med 2004 351 2817 2826 15591335 10.1056/NEJMoa041588

[B92] Barthélémy P Heitz D Mathelin C Polesi H Asmane I Litique V Rob L Bergerat JP Kurtz JE Adjuvant chemotherapy in elderly patients with earlybreast cancer Impact of age and comprehensive geriatric assessment on tumor board proposals Crit Rev Oncol Hematol 2011 79 2 196 204 Aug 20655243 10.1016/j.critrevonc.2010.06.005

