# Supplementary material for: Prognostic factors in elderly patients with breast cancer
Source: BMC Surg. 2013 Oct 8;13(Suppl 2):S2. doi: 10.1186/1471-2482-13-S2-S2 (PMC3851261; doi:10.1186/1471-2482-13-S2-S2)
Supplement: Additional file 1 — Principal studies correlating older ages and prognostic factors. These studies fulfilled the criteria for inclusion [file 1471-2482-13-S2-S2-S1.pdf]

Additional file 1. Principal studies correlating older ages and prognostic factors.

Authors	Year	N	Age	follow up	Aim of the study	Relevant prognostic factors identified and/or conclusions
Ali et al,	2011	14048	>50 (40% >70)	5 and 10 yrs	Survival in different age groups	Age >75, lack of screening, deprivation predict poorer survival.
Barthelemy	2011	192	76,7 (70-98)		Impact of age and CGA on prescription of therapy.	Older age is a risk factor of under-treatment
Botteri	2010	12152	52		Relationship between lymph node (LN) involvement and age.	Delayed diagnosis is predictive of LN*.
Bouchardy	2003	407	>80	5 yrs	Determinants and effect of treatment choice.	Pf: age, under-treatment.
Chagpar	2009	4131	60 (27-100)		Adequacy of AND	Increasing age correlates with less adequate AND (<10 LN).

Chagpar	2007	700	76 (>70)		Develop a clinical rule to predict node positivity.	T, G, LVI are predictive of node positivity.
Clough-Gorr	2010	660	>65	5-10 yrs	Survival for Cancer specific CGA in early BC.	≥3 cancer specific CGA predict poor prognosis.
Ejlertsen	2009	15659	All ages	7,7	Significant of LVI	LVI isn't an independent risk factor.
Gajdos	2001	206	>70		Comparing treatment and outcome with younger	Although under-treatment was more frequent in older people, it doesn't affect chance of survival or risk of recurrence.
Gennari	2004	2999	>50		Evaluating relationship between biologic features and treatment choice	Older women have more favorable biological characteristics. Treatment should be chosen on the basis of biologic characteristics, comorbidity, social support, functional status, and patient preferences.
Hartmann	2011	4056	All ages	5 yrs	Diabetes as prognostic factor	Diabetes doubles risk for distant metastases in ER-; diabetes doubles mortality on 5 yrs follow up.

Hieken	2004	104	>65		Utility of SNB in elderly	28% of elderly had ≥1 N+.
Livi	2006	1500	>65<br>(65-87)	8,7 yrs	Evaluating prognostic factors in elderly	For cancer-specific survival (CSS): local relapse, PN status, the type of surgery, radiotherapy, chemotherapy. For local disease-free survival (LDFS): mastectomy, histotype, PN status, and pt status.
Lyman	2003	20799	All ages		Evaluating risk of under treatment	Older ages.
Martelli	2005	219	65-80	5 yr	Randomized trial; survival and axillary relapse in pts undergone to CBS + TMX vs. BCS + AS + TMX	Axillary dissection may be safety omitted in older patients with early stage breast cancer and no palpable axillary nodes since its omission has no discernible effect on breast cancer mortality or overall survival.
Martelli	2011	671	≥70	15	Retrospective analysis comparing elderly patients with early breast cancer who received conservative surgery with or without axillary	Axillary dissection can be omitted.er negative status is associated with significantly greater rates of distant metastases and breast cancer death

					dissection.	
Melicharovà	2010				Test urinary neopterin as marker of comorbidity.	Significantly higher neopterin levels were noted in>70 yrs patients or with two or more comorbid conditions.
Mittendorf	2012	509 + 464			Incorporation of metastasis size in SN in a nomogram.	Size of node metastasis is useful to predict no SN metastasis.
Moghaddam	2010	108			Comparison of nomograms.	Similar results.
Muss	2005	6487	All ages	9.6 yrs	Usefulness of chemotherapy in older vs. younger patients.	Predictive factors of better survival are smaller t, fewer N+, higher doses CHT and TMX : more (0.5%) therapy related deaths.
Owusu	2008	961			Factors predicting interruption in HT.	Predicting factors are age >75 v < 70 years; increase in Charlson Comorbidity Index at 3 years from diagnosis; increase in the number of cardiopulmonary comorbidities at 3 years; indeterminate ER status v ER-positive status; breast-conserving surgery (BCS) without radiotherapy vs.

						mastectomy.
Pal	2008				Validation of nomograms.	
Patnaik	2011	64034	≥66	5 yrs		Comorbidities worsen survival outcomes like or more than later stage at diagnosis. Myocardial infarction, peripheral vascular disease, stroke, chronic obstructive pulmonary disease, and diabetes determine outcome as shifting the stage by one from I-II to II-III; dementia, chronic renal failure, and liver disease shift the outcome by two stages (from I-II to III-IV).
Phua	2010	137	>70	5 yrs	Retrospective study: OS, CSS; prognostic factors.	Performance status, T3-4 tumor, M +, G and ER status were independent prognostic factors for OS. For CSS : T4 tumor, M+ and ER status.
Poltinnikov	2006	153	≥70	55mo	Cause specific survival (CSS), (OS), and combined nodal and distant failure (NDF). Her-2 neu positivity	Her-2 neu + predicts development of NDF and lower CSS.

Quaglia	2011	1081	All ages	5 yrs	Effect of age and socio-economic status (SES) on CSS	≥70 in lower SES had 58% CSS vs. 91% of younger and wealthier.
Ramjeesingh	2009	397	All ages		Prediction of metastases in sentinel node; validation of nomogram	Multifocality and LVI predictive for SN +; MSKCC nomogram overrated the risk of NSN metastases.
Richards	1999	2022	All ages		Influence of delay in diagnosis on OS and CSS	Longer delays are associated with worse survival. At 10 years from onset of symptoms a difference in CSS was observed between patients with delays of less or more than 12 weeks (57% vs. 537%). At 20 years, the difference in CSS was 48% vs. 33%.
Satariano and Ragland	1994	936	All ages	3 yrs	Effect of comorbidity on survival	3 comorbidity determine a 4-fold higher all-causes mortality.
Schonberg	2010	49616	≥67	5,6 (2-13) yrs	Variations in breast cancer tumor characteristics, initial treatments received, and survival among	Age >80 increases risk to die from BC. Mastectomy, BCS alone, or no surgery are predictive of poorer prognosis respect to BCS+RT. Chemotherapy was associated with a

					women age 80 to 84, 85 to 89, and ≥90 years with early-stage (stage I or II) breast cancer compared with younger women (age 67 to 79 years).	significant reduction in mortality for women age 67 to 79 years (AHR, 0.6; range, 0.5 to 0.8) and an increased risk of mortality for women age ≥80 years (AHR, 1.5; range, 0.9 to 2.3) that did not achieve statistical significance.
Schrauder	2011	4056	All age. 3027 post-menopausal, mean age 57,6	1-16 yrs	Influence of diabetes on survival, distant metastasis-free survival and local recurrence free survival in relation to common tumor and patient characteristics.	Patients with DM were significantly older (mean 67,4) at diagnosis of BC and presented with a higher tumor stage. Mortality following BC was significantly higher among women with DM compared to BC patients without DM (HR 1.90; 95% CI 1.49–2.48).
Silliman	2009	1859	≥65	10 yrs	Linking variations in treatment and follow-up care to variations in outcomes.	≥75 years of age, with more comorbidity, low or intermediate risk of recurrence are more frequently associated with non-standard primary therapy, particularly BCS without rt. BCS without RT was associated with : increased rate of recurrence, second primary breast cancer and breast cancer mortality. Among hr + tumors group, less than one year of tamoxifen was associated with a higher rate of

						recurrence, second primary BC and lower CSS than five years or more. ≥ 80 years of age and comorbid conditions reduce likelihood of yearly mammograms. Each additional surveillance mammogram was associated with a reduction in the odds of breast cancer mortality.
Smidt	2005	229			Validation of nomogram	The nomogram provides a fairly accurate prediction of the probability of non-SLN metastases.
van de Water	2012	9766	All age	5.1 yrs	Association between age at diagnosis and breast cancer outcome in postmenopausal women with hormone receptor–positive breast cancer	Higher risk of disease-specific mortality with increasing age (reference standard, patients <65 years [hazard ratio {HR} for patients aged 65-74 years, 1.12; 95% CI, 0.94-1.34; hr for patients aged ≥75 years, 1.66; 95% CI, 1.34-2.06; *p* <.001]).
van den Hoven	2010	168			Validation of nomogram	Performance of the MSKCC nomogram was insufficient.
Van Zee	2003	702 + 373			Validation of nomogram	Pathological size, tumor type and nuclear grade, lymphovascular invasion, multifocality, and estrogen-

						receptor status of the primary tumor; method of detection of SLN metastases; number of positive SLNs; and number of negative SLNs are criteria useful to develop a nomogram to easily and accurately calculate the likelihood of having additional, non-SLN metastases for an individual patient.
Wagner	2011	818			Effect of time to surgery on tumor growth by comparing initial imaging and pathologic tumor size	Modest time intervals (mean 21 days, range 1-132) from imaging to surgery are not significantly associated with change in tumor size.
Wang	2010	31298	All ages		Pattern of breast cancer surgery after adjusting for other major prognostic factors in relation to patient age.	After adjusting for histologic grade, number of cancers, tumor size, tumor location, the EIC presence (yes or no), and LN status (+ or −), compared with the group aged 51–70 years (the reference group), the group aged >70 years had only half the chance of receiving BCS (OR=0.498, 95% ci: 0.455–0.545).there was no difference between women aged 51–70 years and those aged >70 years in

						tumor histologic grade, prevalence of LN+, and LVI although higher t in women aged >70 years.
Williams	2008	4222	≥65		Relationship between wealth and screening mammography use in older women according to life expectancy	Financial resources determine access to screening mammography more than the possible benefit. Poorer older women with favorable prognoses are at risk of not receiving screening mammography when they are likely to benefit.
Wyld et al,	2004	378	55-98		Investigate reasons for failure in improving outcome in older women	Stage at presentation of breast cancer is more advanced in older women and that this difference may be largely due to lack of mammographic screening. Thus, their treatment more frequently falls outside of agreed national and local guidelines.
Yood	2008	1837	≥65		To compare the effect of primary breast cancer therapy (breast-conserving surgery [BCS] without radiation therapy [RT], BCS plus RT,	After adjusting for age, race and ethnicity, baseline Charlson Comorbidity Index category, tumor size, number of positive lymph nodes, receptor status, and histologic grade, women receiving BCS without RT were more likely

					and mastectomy) on mortality, and the additional effect of tamoxifen among those with hormone-responsive tumors	to die of breast cancer (HR=2.19, 95% CI, 1.51 to 3.18) than women receiving mastectomy (adjusted rate difference = 50%). Women receiving BCS+RT had a rate of breast cancer death similar to that of patients receiving mastectomy (HR=1.08, 95% CI, 0.79 to 1.48; adjusted rate difference=0.4%). Tamoxifen for less than 1 year was associated with a higher rate of death compared with 5 years or more, (HR=6.26, 95% CI, 3.10 to 12.64). The survival benefit from tamoxifen increased with increasing therapy duration.
