# Supplementary material for: Prognostic factors in elderly patients with breast cancer
Source: BMC Surg. 2013 Oct 8;13(Suppl 2):S2. doi: 10.1186/1471-2482-13-S2-S2 (PMC3851261; doi:10.1186/1471-2482-13-S2-S2)
Supplement: Additional file 2 — Other studies consulted and cited even not fulfilling criteria. The studies listed in this section were relevant, though not fulfilling criteria (i.e.: consensus conference, validation study, studies including all ages patients or not aimed to survival.). The aim of the study is descripted too. [file 1471-2482-13-S2-S2-S2.pdf]

Additional file 2. Other studies consulted and cited even not fulfilling criteria.

Critical points	Authors	Year	Aim of the study
consensus conference, guidelines and recommendation	Fitzgibbons	2000	consensus conference
	Lyman	2005	guidelines
	Kimmick &Muss	2001	recommendation
	Audisio	2004	recommendation
	Biganzoli	2012	recommendation
no survival informations	Durbecq	2008	influence of age on the incidence of BC molecular subtypes
incidental or no age criteria	Alran	2007	validation study,
	Klar	2008	validation of nomogram
	Kohrt	2008	validation of nomogram
	Pal	2008	validation of nomograms
	Gur	2010	validation study of nomogram
	Melicharovà	2010	test urinary neopterin as marker of comorbidity
	van den	2010	validation of nomogram

Hoven		
Wagner	2011	effect of time to surgery on tumor growth by comparing initial imaging and pathologic tumor size
Mittendorf	2012	incorporation of metastasis size in SN in a nomogram
Sasada	2012	validation of nomogram
Charlson	1994	impact of comorbidity and description of score system
Carter	1989	impact of T and N on survival
Van Zee	2003	validation of nomogram
Somner	2004	establish recommendations for the number of lymph nodes to be recovered from each AND specimen
Barranger	2005	validation study
Ramirez	1999	Prediction of delayed diagnosis
Smidt	2005	validation of nomogram
Poltinnikov	2006	cause specific survival (CSS), (OS), and combined nodal and distant failure (NDF). Her-2 neu positivity
Ponzone	2007	validation of nomogram
Coutant	2008	validation study of nomogram
Di Vita	2010	Prognostic factors
Ahn	2011	Lymph node ratio and pN staging
Hidar	2011	validation study of nomogram
Andersson	2012	validation study
Cappellani	2012	review
Paik	2004	Validation study (multigene essay)
Albrand &	2008	review

	Terret		
	Leonard	2011	efficacy of CHT in elderly
no survival information nor predictive factors	Repetto	2002	utility of geriatric assessment
	Lavelle et al	2007	rating non standard treatment in elderly
	Chatzidaki	2011	Histologic characteristic
not in English	Solej	2007	Validation study (surgery)
small sample,	Yildirim	2008	To test the effect of the immune response on the outcome of patients with cancer
	Orsoni	2011	validation of nomogram
	Osborn	2011	efficacy of primary HT
small sample, incidental age criteria	D'Eredità	2010	validation study of nomogram
	Lowery	2011	compare prognostic biomarkers between interval and screen-detected breast cancers overall, and according to age at diagnosis and familial risk
	Unal	2009	validation of nomogram in patients receiving neoadjuvant CHT (NCT)

study on metastatic cases	Janssen-Heijnen	2007	survival
	Jung	2012	survival
